# The effect of a visual illusion and self-controlled practice on motor learning in children at risk for developmental coordination disorder

**DOI:** 10.1038/s41598-024-63387-z

**Published:** 2024-05-30

**Authors:** Reyhane Shahbaz, Esmaeel Saemi, Mohammadreza Doustan, Jennifer A. Hogg, Jed A. Diekfuss

**Affiliations:** 1 Department of Motor Behavior and Sport Psychology, Faculty of Sport Sciences, Shahid Chamran University of Ahvaz, Ahvaz, Iran; 2 Department of Health and Human Performance, The University of Tennessee Chattanooga, Chattanooga, TN USA; 3 Emory Sports Performance And Research Center (SPARC), Flowery Branch, GA USA; 4 Emory Sports Medicine Center, Atlanta, GA USA; 5 Department of Orthopaedics, Emory University School of Medicine, Atlanta, GA USA

**Keywords:** Enhanced expectancies, Autonomy support, Motor learning, Ebbinghaus illusion, DCD, Psychology, Learning and memory

## Abstract

Numerous efforts have been made to test the OPTIMAL theory of motor learning in healthy children and adult populations. However, only a small number of studies have tested this theory in children with cognitive-motor disorders, such as developmental coordination disorder (DCD). The present study aims to examine the individual and additive effects of a visual illusion and self-controlled practice on a golf putting task in children at risk for DCD based on the OPTIMAL theory. Forty children at risk for DCD (mean age = 8.57 ± 1.05 years) were randomly assigned to four experimental groups (1—small visual illusion + self-controlled practice; 2—big visual illusion + self-controlled practice; 3—small visual illusion + yoked; 4—big visual illusion + yoked). Following 12 pretest trials of a golf putting task, the participants completed 5 blocks of 12 trials of practice on the first day. A retention test (12 trials) and a transfer dual-task test (12 trials) were conducted on the second day. The results indicated that in retention test the big visual illusion + self-controlled practice group was significantly better than the small visual illusion + yoked group (p = 0.01), while there was not any other significant difference between groups at retention test as well as between all groups at practice phase and transfer test (p > 0.05 for all comparisons). In other words, an additive effect has been observed just in the retention test but not the practice phase as well as transfer test. In general, the results of this study support the OPTIMAL theory of motor learning in children at risk for DCD and suggests to all educators who work with these children to use the combination of the visual illusion with self-controlled practice to improve the motor learning of children at risk for DCD.

## Introduction

Many children, despite apparently typical physical development, retain deficiencies in motor behavior^1^. The term developmental coordination disorder (DCD) is used to describe motor problems in children^2,3^. The American Psychiatric Association defines DCD as a neurodevelopmental impairment affecting 5–6% of children, which remarkably influences a child’s ability to learn and perform daily tasks such as self-care and school-work^2^.

Children with DCD face difficulties in gross and/or fine motor skills^4^ and have problems in performing cognitive-motor actions that require eye-hand or hand-leg coordination^5^. Studies have shown that visual information takes precedence over all sensory information received by children with DCD. In other words, when performing daily motor tasks, these children are largely more dependent on the visual information they receive compared to other forms of sensory information such as auditory and tactile^6–8^. Thus, one probable reason behind the motor control deficiencies in children with DCD can be attributed to their strong dependence on visual information, neglecting other forms of sensory information, such as proprioception or vestibular information^7^. In other words, studies have shown that children with DCD rely more on their visual information to perform motor skills and daily tasks, and the capability of using other sources of information is less than that of typically developing children, While children with DCD may be able to improve their abilitiy to use auditory and tactile information, maximizing the use of visual cues may be a promising area for improvement. Cnsidering the limited research in this field, conducting research that deals with the manipulation of visual information in DCD children is necessary.

Wulf and Lewthwaite^9^ have proposed the OPTIMAL (Optimizing Performance through Intrinsic Motivation and Attention for Learning) theory of motor learning. The theory is based on three major factors that influence motor skill learning and acquisition. The first factor is external focus of attention which facilitates learning when compared with an internal focus of attention^10,11^. The second factor is enhanced expectancies; motor learning is facilitated when the learner has a greater expectation for their own success^12–14^. The third factor is self-controlled practice (autonomy support), in which the giving the learning control over some aspect of their learning conditions leads to improved acquisition and retention of a motor task^15,16^. However, although the external focus of attention is one of the most important factors in the OPTIMAL theory of motor learning and it is known to increase motor learning and performance in children with developmental disorders and especially children with DCD^17^, there is less research on the two others factors of the OPTIMAL theory: enhanced expectancies and self-controlled practice. Therefore, in the current study, the researchers focused on the second and third factors and their additive effect in children at risk for DCD.

Up to date, numerous efforts have been made to test the OPTIMAL theory on learning, particularly in typical learners. Support for the OPTIMAL theory has been reported in healthy adult^18–21^ and pediatric populations^22^. Indeed, autonomy support and self-controlled practice, external focus of attention, and enhanced expectancies have been shown to be more effective collectively than when tested individually for adults completing a ball throwing task^19^. Similar findings have been demonstrated for typically developing children^20^; under combined self-controlled practice and external focus manipulations, children experienced higher levels of motor learning in a bowling task.

As a primary tenet of the OPTIMAL theory, enhanced expectancies can be implemented in a variety of ways. Some studies increased enhanced expectancies by providing feedback only following successful trials^14^; others provided augmented social-comparative feedback about an individual’s superior performance over other performers^23,24^; still others presented an easy criterion for completing a task compared to a more difficult criterion, thereby reducing the perceived task difficulty^13,25^. Manipulating a perceived target size can be accomplished through the use of visual illusions^12,26,27^. Visual illusions take advantage of an error in the visual system that can naively misperceive real conditions^28^. One type of visual illusion is the Ebbinghaus illusion, which consists of two sets of circles, each set having one central circle surrounded by either larger or smaller circles. The juxtaposition of the two sets results in an illusion that the two central circles are different sizes; individuals perceive the circle surrounded by smaller circles to be larger than the circle surrounded by larger circles, when in reality they are the same size^29^. Many studies have incorporated visual illusions into the study of motor learning and have reported positive effects^12,27,29,30^. Specifically, when the target is perceived to be larger than its actual size, motor performance and learning improve in adults^12^ and in children^26^.

Although most of these studies have pointed to positive effects of enhanced expectancy on motor learning, few studies have examined individuals with disorders, particularly children with DCD. For example, Razeghi et al.^31^ demonstrated that manipulating perceived task difficulty through a visual illusion can improve motor learning for golf putting in individuals with autism.

Based on some related studies, children with DCD will be more affected by visual bias than age- and sex-matched typically developing children^8^. In other words, research findings show that there is a positive relationship between visual bias compared to other information sources such as tactile bias and poor motor performance in children with DCD^8^. In addition, since these children are less sensitive than adults to Ebbinghaus illusions^32^ and given the considerable dependence of children with DCD on visual sensory information for performing different cognitive and motor skills^6–8^, and also, the difference between children with DCD and typical children responding to visual illusions^8^, studying the effects of enhanced expectancy by manipulating perceived task difficulty through the use of visual illusions in these children may yield promising information.

On the other hand, autonomy support, or self-controlled practice, operationalized as giving the learner control over their learning environment, can lead to robust motor learning^20,33–36^. Learners with greater independence in their training sessions exhibit greater motivation and become actively engaged in learning, and this in turn leads to improved skill learning^16^ For instance, studies have shown that learners under self-controlled practice conditions experienced greater learning than under yoked practice conditions^33–36^. This superiority has also been observed in children^20^. However, there are few studies that examined self-controlled practice in individuals with disorders, particularly in children with DCD, a necessary step toward developing effective motor learning strategies for this population.

There are two theories to explain the mechanism between self-controlled practice and motor learning^35^. The first explanation is motivational, which attributes improved learning and performance during self-controlled practice to satisfaction of an essential need for autonomy in individuals^37^. The second one links improved learning and performance under self-controlled practice to an information processing approach, suggesting that due to facilitated error estimation under self-controlled practice, learning can improve as a result of enhanced information processing^38^.

Studies in children with DCD have found that these children have little motor experience, are unfamiliar with motor tasks, have problems in focusing their attention^39^ and excessively rely on visual information^6–8^ during motor skill performance. Therefore, engaging children with DCD through the development of targeted strategies likely to be effective in this population can improve motor performance, ultimately resulting in an improved quality of life.

Little emphasis has been placed on incorporating enhanced expectancy and self-controlled practice into motor learning improvement programs in children with DCD. Hence, the present study attempts to answer the question of whether a combination of practice techniques using a visual illusion paradigm to enhance expectancies and self-controlled practice to foster autonomy support can improve motor learning in children at risk for DCD beyond the isolated effects of either. We hypothesized that individual and additive effects of a visual illusion-based enhanced expectancy manipulation and self-controlled practice will improve golf putting in children at risk for DCD.

## Materials and methods

### Participants

The participants in the present study were 40 children aged 7 to 10 (mean age = 8.57 ± 1.05; all right-hand) at risk for DCD identified from 5 local elementary schools using the developmental coordination disorder questionnaire^40^. An a priori sample size was calculated in G*Power. Given the significance level at 0.05, the statistical power of 0.90, the effect size of f = 0.25^17^, and with two independent variables (visual illusion and self-controlled practice) and with a within- and between-subject design, the sample size was calculated at 40 participants. We included individuals who (1) were in the age range 7 to 10; (2) did not have neurological or motor problems; (3) were novice in terms of golf putting skills; (4) were identified as children at risk for DCD based on the questionnaire developed by Wilson et al.^40^; and (5) had a normal IQ (IQ ≥ 70). We excluded individuals who (1) did not have parental consent despite the initial agreement during the experiment; (2) did not attend the retention and transfer tests as scheduled; or (3) experienced any injury during the experiment. Prior to the test, the participants and their parents completed a consent form at the laboratory to indicate their consent for participating in the study. The present study is a quasi-experimental design approved by the university’s Research Ethics Committee (EE/1401.2.24.173107 /scu.ac.ir) and conducted based on the Helsinki protocol.

### Instrument

#### DCD Questionnaire (modified version) (DCDQ; Wilson et al.^38^)

DCDQ is a common measure to identify 4-to-12-year-old children at risk for DCD and has good validity and reliability^41,42^. Salehi et al.^43^ evaluated the retest reliability of the Persian DCDQ at 0.93 and found its correlation with the subtests of relocation (r = 0.65) and manipulation (r = 0.6) of the test of gross motor development -2 (TGMD-2). Exploratory and confirmatory factor analysis showed that the Persian inventory represents a valid and reliable instrument for screening Iranian children^43^. The DCDQ contains 15 items that measure three factors, namely control during movement, fine motor/handwriting, and general coordination. It consists of a teacher and parent form and here we used the parent form. The items are scored on a Likert scale: 1 = “not at all”, 2 = “a little”, 3 = “to some extent”, 4 = “a lot”, and 5 = “very much”^43^. According to the DCDQ scoring guide, children of the age 5 years to 7 years and 11 months scoring 15 to 46 on DCDQ and children of the age 8 years to 9 years and 11 months scoring 15 to 55 on DCDQ are recognized as potentially having DCD^44^.

#### Golf putting skill

**Figure 1:**
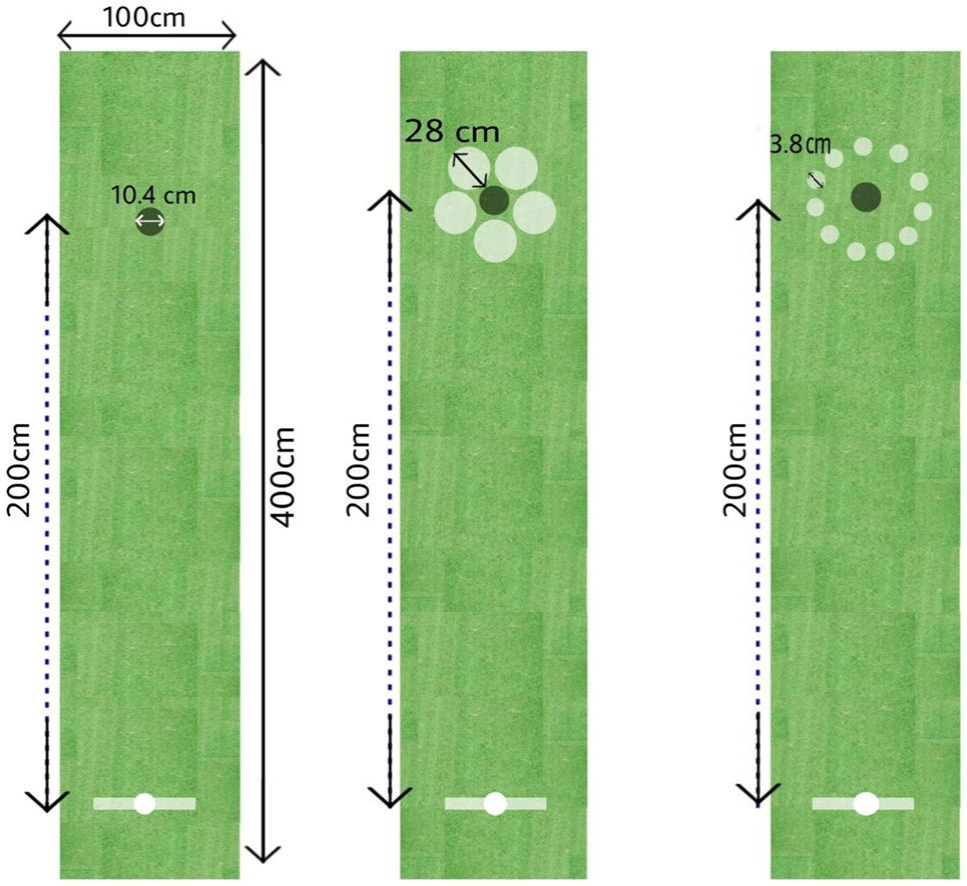
A schematic illustration of the golf putting task for all experimental conditions (normal condition and small or big visual illusion conditions).

The instruments consisted of a piece of artificial-turf indoor green, a golf putter, and a golf ball. The participants used a right-hand putter for children, 75 cm in length, to putt a standard golf ball over a 400 cm by 100 cm turf. The turf contained a target hole (10.4 cm) placed 200 cm from the starting point. The starting point was marked using white tape 5 cm in width placed in the front of the putting individual. Each individual’s score was calculated as radial error (the distance from the ball to the center of the hole) for each trial and recorded in centimeters (Fig. 1).

### Procedure

**Figure 2:**
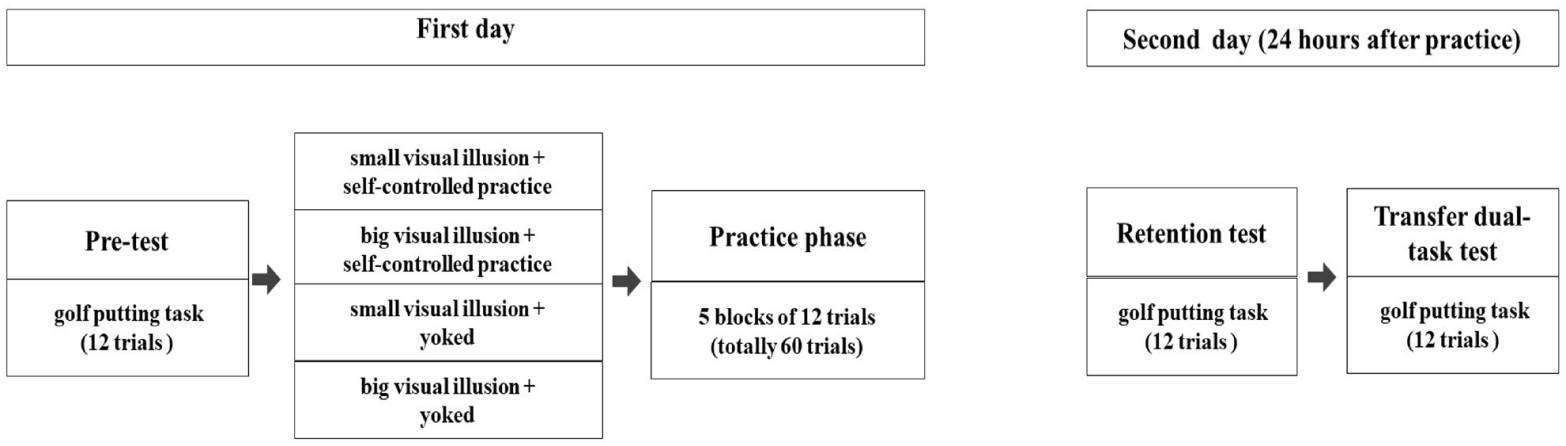
The flowchart of study procedure.

This quasi-experimental study consisted of pretest (first day), practice (first day), retention and transfer tests (second day; Fig. 2). The participants were 40 children (8 females) at risk for DCD identified using DCDQ developed by Wilson et al.^40^ for parents. They were match by gender and then were randomly assigned to four experimental groups (to maintain the ratio between the two genders, boys were randomly assigned to groups first and then girls randomly assigned to groups; 8 boys and 2 girls in each experimental group): (1) small visual illusion + self-controlled practice (N = 10), (2) big visual illusion + self-controlled practice (N = 10), (3) small visual illusion + yoked (N = 10), and (4) big visual illusion + yoked (N = 10). All participants completed 12 trials of the golf putting skill for the pretest under normal conditions (without the illusion). In the practice phase, the participants performed 60 practice trials in 5 blocks of 12 trials each according to their experimental groups. The rest period between practice blocks was about 3 min. Next, 24 h following the last practice block, the participants took part in the retention and transfer tests. For the retention and transfer phases the participants completed 12 practice trials each. The transfer task was a dual-task in which the children completed a secondary cognitive task simultaneous with the golf putting task (countdown numbers task). This was done to assess the children’s level of automaticity^45^.

Under the big visual illusion condition, the hole in the golf turf was surrounded by smaller circlers. The target hole was surrounded by 11 small circles 3.8 cm in diameter and 5 large circles 28 cm in diameter. Thus, based on Ebbinghaus illusion, the target hole would look larger than it actually was in diameter. In contrast, when the target hole is surrounded by larger circles, it looks smaller than it actually is (Fig. 1). In the present study, the participants in the self-controlled practice groups were asked to choose the color of the ball. Allowing learners to choose the color of the ball, despite this choice being irrelevant to task goal, is a common protocol in research based on self-controlled practice^21^. In these studies, the self-controlled group is allowed to choose the color of the ball used for practice. But on the other hand, each learner in the yoked group must use a ball with the same color as a learner from the self-controlled group, exactly during the practice trials. In this way, environmental effects can be isolated from the effects of choice. In the practice phase for the self-controlled practice groups (small visual illusion + self-controlled practice and big visual illusion + self-controlled practice) the participants picked a color from blue, yellow, and red in each trial. However, the participants in the yoked group had to complete the putting task based on the colors chosen by their respective self-controlled practice group^34^. To assess learning level among the participants, retention and transfer tests were conducted 24 h following the completion of the practice sessions. In the retention test, the participants completed 12 trials of golf putting when the target hole was not surrounded by circles (similar to pretest phase; without the illusion) from a 2-m distance. The transfer test was similar to the retention test, only this time the test was conducted under cognitive dual task conditions. The participants had to complete a simultaneously cognitive task involving a number countdown during the golf putting task. In the countdown task, the children were told to count backwards starting from the number 15, in such a way that the child counts the numbers aloud and backwards at the same time as the golf putt is executed (numbers:15, 14, 13, … ). The interval between voicing numbers was 1 s^45^.

### Data analysis

Statistical analysis was conducted in the present study by inspecting means and standard deviations for descriptive statistics together with inferential statistics to assess isolated and additive effects of self-controlled practice and a visual illusion. Specifically, one-way ANOVAs compared demographic characteristics (e.g., age, height and weight) as well as baseline golf putting scores between the four experimental groups. To test acquisition, a 4 (group) by 5 (block; repeated factor) mixed ANOVA was conducted. To test retention and transfer, separate one-way ANOVAs to compare golf-putting scores between the four groups were conducted. Post-hoc testing was conducted as appropriate (Tukey’s HSD post-hoc test). Alpha was set at α = 0.05. The data were statistically analyzed in SPSS 28 and the charts were plotted using Excel 2016.

### Institutional review board statement

The present study is a quasi-experimental design approved by the university’s Research Ethics Committee (EE/1401.2.24.173107/scu.ac.ir) and conducted based on the Helsinki protocol.

### Informed consent

Informed consent was obtained from all parents of children involved in the study.

## Results

The initial findings indicated that our data were normally distributed. The findings also showed that all four groups were similar in terms of participants’ individual characteristics and baseline golf putting skill (Table 1).

**Table 1:** Participants’ individual characteristics.

Individual characteristics	Groups (M ± SD)				p-value
	SVI-SC	BVI-SC	SVI-Y	BVI-Y	
N	10	10	10	10	-
Sex	8 M, 2 F	8 M, 2 F	8 M, 2 F	8 M, 2 F	
Age (year)	8.50 ± 0.97	8.90 ± 1.19	8.50 ± 0.85	8.40 ± 1.26	0.74
Height (cm)	119.00 ± 18.81	125.00 ± 19.48	124.80 ± 14.62	130.90 ± 13.14	0.47
Weight (kg)	29.50 ± 4.14	32.30 ± 9.34	34.10 ± 7.40	34.00 ± 7.98	0.48
Golf putting accuracy (pretest, cm)	40.94 ± 5.33	39.87 ± 6.30	39.61 ± 4.15	39.98 ± 4.87	0.91

M: male; F: female; SVI-SC: small visual illusion + self-controlled practice group; BIV-SC: big visual illusion + self-controlled practice group; SVI-Y: small visual illusion + yoked group; BVI-Y: big visual illusion + yoked group.

### Practice

To assess the effects of visual illusion and self-controlled practice during the practice stage, a 4 (group) by 5 (block; repeated factor) mixed ANOVA was conducted. The initial findings confirmed Mauchly’s assumption of sphericity (p > 0.05), so no correction was made. The multivariate omnibus was not significant (Wilks’ λ = 0.809, F(4,33) = 1.945, p = 0.13) (Fig. 2).

### Retention

To test retention, a one-way ANOVA was conducted to assess golf-putting scores between groups at the post-test time point. It was observed a significant main effect for group (F(3, 36) = 3.86, p = 0.017, η^2^ = 0.24), wherein the big illusion + self-controlled practice group demonstrated lower error than the small illusion + yoked group (mean difference = 9.96 cm; pairwise p = 0.01) at the retention time point. No other significant effect was observed between groups (Fig. 2).

### Transfer

**Figure 3:**
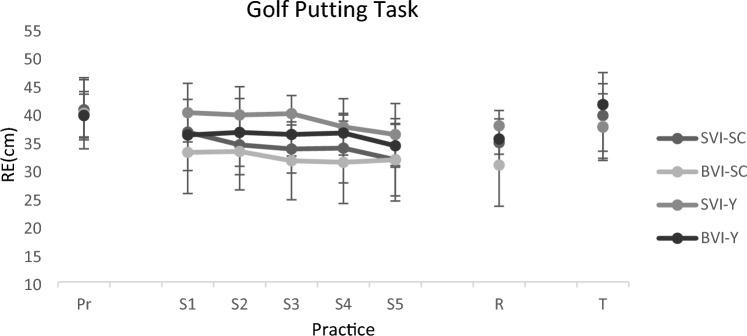
Golf putting accuracy for all experimental groups during pretest, practice, retention as well as transfer. Error bars represent standard deviation. As can be seen, the experimental groups are similar in the practice phase as well as the transfer test, but in the retention test, it can be seen that the big visual illusion + self-controlled practice group is significantly better than the small visual illusion + yoked group while there are no significant difference between other groups. SVI-SC: small visual illusion + self-controlled practice group, BIV-SC: big visual illusion + self-controlled practice group; SVI-Y: small visual illusion + yoked group; BVI-Y: big visual illusion + yoked group; RE: radial error.

To test transfer, a one-way ANOVA was conducted to assess golf-putting scores between groups during the dual-task transfer task. No significant effect was observed (F(3) = 1.12, p = 0.36, η^2^ = 0.09) (Fig. 3).

## Discussion

The present study was designed with the aim of testing the OPTIMAL theory of motor learning^9^ in children at risk for DCD and it was intended to investigate the effect of visual illusion (enhanced expectancies) and self-controlled practice (autonomy support) on motor learning and performance of the golf putting task in children at risk for DCD. The results of the present study supported the OPTIMAL theory of motor learning in children at risk for DCD and showed that in retention test, the combination of big visual illusion and self-controlled practice improve motor learning of the golf putting task in children at risk for DCD. However, these results were not observed in practice phase as well as transfer test under dual task conditions. In other words, these results indicated that the additive effect of visual illusion and self-controlled practice is more positive than both their individual effects.

Our findings showed that practicing with a big visual illusion can enhance motor learning compared to a small visual illusion. This means that our findings point to the effectiveness of enhanced expectancies in improvement of motor learning in children at risk for DCD. The findings supported our hypothesis on the effectiveness of a visual illusion to alter perceived task difficulty for learning in children at risk for DCD and are in line with major findings of other studies in this domain^12,26,27,29,31^. For example, Chauvel et al.^12^ supports the results of the current study, showing that practicing a golf putting task in adults can improve motor learning in conditions of large visual illusions compared with small visual illusions. Altering perceived task difficulty through visual illusions has been supported for use in children^26,31^ and adults^12,30^ for motor skill learning. Furthermore, Wood et al.^46^ showed that manipulating visual illusion and perception of a target to be larger than its actual size can improve motor learning in golfers due to increased fixation time and quiet eye duration in learners during putting skill training. A quiet eye is defined as the final fixation or gaze tracking on a specific point right before starting to move and is typically quantified as remaining within 3° of visual angle for at least 100 ms^47,48^. According to research, longer duration of quiet eye is related to the more visual perception as well as better motor performance^47,48^.

Our findings are further supported by a study that demonstrated increased self-efficacy in children following the learning of a skill via an Ebbinghaus illusion^26^. They found that self-efficacy increased in a group of children who perceived the hole as larger. This group also exhibited more accurate performance during the retention test. Similar findings were also reported in children with autism, suggesting that big visual illusion can enhance motor learning and quiet eye duration in autistic children^31^. These results are consistent with our findings which showed that children with DCD can attain higher levels of motor learning under a big visual illusion condition (i.e., when the target is perceived to be larger than its actual size). Our findings, together with these previous studies, support the motivational aspects of the OPTIMAL theory proposed by Wulf and Lewthwaite^9^ who proposed that enhanced expectancies can facilitate effective goal-action coupling^9^, reduce self- focused attention during execution of a skill^49^, improve motivation^50^, and enhance self-efficacy of learners^26^, leading to improved motor learning. Some studies have shown that children with DCD have problems in focusing their attention^39^ and display excessive reliance on their visual information^6–8^ while performing a motor skill. In the present study, it appears that increasing the perceived size of the golf target was able to leverage their reliance on vision to improve motor performance.

There are of course a number of studies in this domain which, inconsistent with our findings, failed to report the effects of visual illusion on motor learning^26^ or in contrast to our findings, reported that a small visual illusion is better than big visual illusion in terms of facilitating motor learning^51^. For example, a study of enhanced expectancies in skilled shooters found that while big visual illusions improved motor performance during acquisition, in a delayed retention test no difference was found between big and small illusion groups^26^. They explained the ineffectiveness of visual illusion in long-term learning by inferring a ceiling effect, arguing that skilled participants acquired a form of high-level automatic motor program due to years of training and presence in international competitions for professional shooters. Conversely, others have presented different results, showing that small visual illusions are more beneficial than big visual illusions for improving motor performance^51^. They argued that when faced with a smaller target, learners are forced to exhibit greater accuracy. They asked participants to practice an aiming task (e.g., marble shooting) with either a big visual illusion or a small visual illusion. They found that the participants in the smaller visual illusion group experienced improvement in their performance from pretest to posttest while the bigger visual illusion group did not exhibit any progress. Interestingly, the control group exhibited some progress from pretest to posttest. They also found that small visual illusions improve motor performance while big illusions do not. In Cañal-Bruland et al. study^51^, it was found that small visual illusion improves motor performance, but in the present study, it was found that large visual illusion improves motor learning. Due to the fact that the motor task (marble-shooting task versus golf putting task) and participants were different (healthy adults versus children with DCD ), it may not be possible to compare directly these two studies. In addition, considering that children with DCD are more affected by the visual bias caused by visual illusions than typical children^8^, it seems that these children experience more visual perception than typical children in the condition of visual illusion. While in the condition of visual illusion, these children experience far less visual perception, this difference in visual perception in two situations (big illusion vs. small illusion) has improved the motor learning of children with DCD. Considering that some studies have shown the positive effects of large visual illusions^12,27,29,31^ and some positive effects of small visual illusions^51^ and some no difernce^26^, it is suggested that more research be done in this field so that it can help us to better understand the impact of visual illusions on motor learning and performance contributes to different motor tasks as well as in different participants.

Our findings also demonstrated that self-controlled practice was superior to yoked practice during retention. These findings supporte our hypothesis about the positive effects of self-controlled practice on motor learning improvement in children with DCD and are consistent with most studies on self-controlled practice^33–36^. These studies have largely shown that providing learners with choice during motor skills practice can facilitate motor learning^36,52^. For instance, Iwatsuki et al.^52^ demonstrated that providing performers with choice can enhance their motor performance compared to a yoked condition.

Given the motivational approach and based on the theory of autonomy^37^, training under self-controlled practice conditions can create an appropriate environment for enhancing learning and individual performance because these conditions help individuals meet their need for autonomy^35^. Based on the OPTIMAL theory of motor learning^9^, practice conditions that give learners options for choice supports their need for independence and therefore increases their intrinsic motivation. This increased motivation can eventually result in improved motor learning. In addition, self-controlled conditions can increase perceived competence which is another basic psychological need with positive direct effects on intrinsic motivation. Studies in this area have supported this claim by showing that self-controlled practice can facilitate motor learning by increasing perceived competence and self-efficacy^53^. Thus, it seems that participants in the present study likely derived motivation from their choice of golf ball color for executing a golf putting skill, which supported a basic psychological need and resulted in improved golf-putting performance.

In line with some research done in this field^18–20,54–57^, the results of the present study also show the additive effect visual illusion and self-controlled practice have on motor learning and performance in children with DCD and support the predictions by OPTIMAL theory of motor learning in children with DCD. All these studies^18–20,26,54–57^, showed that combining motivational (enhanced expectancies and autonomy support) or attentional factors (external focus) and including them in practice sections can create higher motor learning for both children^26^ and adults^19,55^ as well as older adults^54^. For example, Ghorbani^18^ showed that participants exhibited improved motor learning when learning a dart-throwing skill when placed in a combined condition of enhanced expectancy and self-controlled practice (additive effect). By confirming the OPTIMAL theory of motor learning, the authors attributed this superiority to the increase in learner's self-efficacy. Based on the OPTIMAL theory of motor learning^9^, when these motivational and attentional factors are put together, they can optimize the learning environment for the learner through goal-action coupling as well as reduce self-focus and also increase focus on the task. It seems that in the current study, children with DCD were able to learn from this rich environment (the environment in which the practice program is designed based on the two motivational dimensions of visual illusion and self-controlled practice) and experienced improved performance in a golf-putting task. Although the results of the present study show the additive effect of visual illusion and self-controlled practice are supported by other studies^18–20,26,54–58^, not all studies show these additive effects^27^. For example, Bahrami et al.^27^ could not show this additive effect and only showed that both visual illusion and external focus of attention variables have a positive individual effect on dart throwing learning, but they do not have a positive additve effect. Makaruk et al.^59^ also demonstrated the individual effects of the two variables of self-controlled practice and external focus of attention on penalty kicking accuracy in moderately skilled participants but could not report the additive effect of these two variables. The difference between the findings of the two mentioned studies and the results of the present research can be due to the fact that both of the mentioned studies were conducted on novice and moderately skilled adults with two different skills (dart throwing and penalty kicks), while our study was in children at risk for DCD during a golf putting task. Forthermore, both of these mentioned studies^27,59^, in addition to the motivational dimension of self-controlled practice the OPTIMAL theory, had the dimension of attention (external focus) and their results showed that the additive effect of self-controlled practice and external focus of attention cannot affect motor learning. While in the present study, the additive effect of the two motivational dimensions of the OPTIMAL theory, i.e. enhanced expectancies and self-controlled practice, have been investigated and its positive effects have been observed. Therefore, another reason for the difference between the findings of the present study and the two mentioned studies^27,59^ can be related to the difference between the motivational dimension of enhanced expectancies and the dimension of attention (external focus).

Our study had a number of limitations. For example, absence of a control group for comparison to our small and big visual illusion groups can be viewed as one such limitation that should be addressed by future studies. While the use of a control group would better elucidate the isolated effects, not having a control group in this study does not influence the observed additive effect. Another limitation of the current study was the lack of typical participants, considering the possible differences between children with DCD and typical children in target perception in visual illusion conditions^8^, it is suggested that future research could examine both groups of children with and without DCD for more clarification. Moreover, we did not account for a number of secondary variables including self-efficacy, intrinsic motivation, and perceived competence which may have enhanced our findings. Also, according to some studies^60,61^, up to 50% of children with DCD have comorbid disorders such as ADHD. Due to the fact that in the present study, this was not measured, therefore, it is suggested to other researchers to consider this limitation and to select only children who only have DCD and not other related disorders such as ADHD.

In addition, according to the OPTIMAL theory, the three factors of external focus of attention, enhanced expectancies and self-controlled practice can enhance the learning environment^9^. In this research, only two factors of enhanced expectancies and self-controlled practice were examined. Due to the additive influence of these three factors, it is suggested that future research examine all three simultaneously in children with DCD to test their additive effect. Fourthermore, the protocol and motor task used in the present study was the golf putting task, which was selected based on some previous similar research in children with developmental disorders^31^. Considering that this case can affect the generalizability of the results of the present study, it is suggested to future similar stusies use other functional/generalisable motor skills that are often impacted in children with DCD to increase the generalizability of thier findings.

## Conclusions

Our findings can have important implications and applications for learning and education environments, particularly for environments that involve children with disorders like DCD. For example, these findings can help sports coaches as well as physical education teachers who work with children at risk for DCD to provide a fruitful environment for increasing the motor learning of these children by simultaneously using enhanced expectancies (applying a big visual illusion) and self-controlled practice (giving the right to choose the color of the ball in ball sports).

## Data Availability

The datasets used and/or analysed during the current study available from the corresponding author on reasonable request.
